# Method for determining predictor factor for worse outcomes in kidney transplant recipients infected with coronavirus disease 2019 in a systematic review and meta-analysis research

**DOI:** 10.1016/j.mex.2023.102250

**Published:** 2023-06-11

**Authors:** Gede Wirya Kusuma Duarsa, Ronald Sugianto, I Gusti Agung Ayu Andra Yusari, Pande Made Wisnu Tirtayasa, Gerhard Reinaldi Situmorang, Nur Rasyid, Arry Rodjani, Besut Daryanto, Kurnia Penta Seputra, Paksi Satyagraha

**Affiliations:** a Department of Urology, Faculty of Medicine, Universitas Udayana, Prof. Dr. I.G.N.G Ngoerah General Hospital, Bali, Indonesia; b Medical Doctor Study Program, Faculty of Medicine, Universitas Udayana, Bali, Indonesia; c Department of Urology, Faculty of Medicine, Universitas Udayana, Universitas Udayana Teaching Hospital, Bali, Indonesia; d Department of Urology, Faculty of Medicine, Universitas Indonesia, Cipto Mangunkusumo National Referral Hospital, Jakarta, Indonesia; e Department of Urology, Faculty of Medicine, Universitas Brawijaya, Saiful Anwar General Hospital, Malang, Indonesia

**Keywords:** Method, Kidney transplant, COVID-19, Risk factor, Outcome, Systematic review, Meta-analysis, PRISMA systematic reviews and Forest Plot Meta-analysis to determine predictor factor for worse outcome

## Abstract

The systematic review and meta-analysis were conducted for COVID-19 infections in kidney transplant patients. Recent research on this topic was still scarce and limited meta-analysis research discussion, specific to some risks or treatment in kidney transplantation patients with COVID-19 infection. Therefore, this article demonstrated the fundamental steps to conducting systematic review and meta-analysis studies to derive a pooled estimate of predictor factors of worse outcomes in kidney transplant patients with positive for the SARS-CoV- 2 test

• PICOT Framework to determine the research scope
• PRISMA strategy for study selection
• Forest Plot for meta-analysis study

## Graphical abstract

**Figure:**
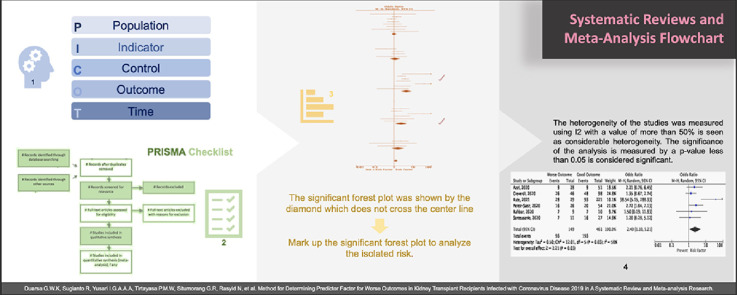

Specifications table

**Table:** 

Subject area:	Medicine and Dentistry
More specific subject area:	Transplantation
Name of your method:	PRISMA systematic reviews and Forest Plot Meta-analysis to determine predictor factor for worse outcome
Name and reference of original method:	1. PICO Framework(1) 2. PRISMA Guildeline(2)
Resource availability:	1. DOI: 10.1186/1472–6947–7-16 2. http://dx.doi.org/10.1136/bmj.n71

**Method details**

## Background

The systematic review and meta-analysis were conducted for COVID-19 infections in kidney transplant patients. Recent research on this topic was still scarce and limited meta-analysis research discussion, specific to some risks or treatment in kidney transplantation patients with COVID-19 infection [3,4]. Therefore, this meta-analysis study aims to determine which predictors, in general, may lead to poor outcomes in kidney transplant patients with COVID-19. To determine the research scope, the authors applied the framework of Population, Indicator, Comparison, Outcome, and Time (PICOT) [1] Table 1.

**Table 1:** PICOT Framework [1].

Framework	Authors Application
Population	Patient underwent kidney transplant before COVID-19 pandemic and suffered from COVID-19 infection
Indicator	Risk Factor associated with patient's characteristics, kidney profile, comorbid, prior medication, symptoms, and laboratory markers
Comparison	Patients without any risk factors
Outcome(s)	In-hospital patients with more severe conditions or death.
Timing	Measured until the first decision of clinical improving or worsening

### Literature search

The authors conducted the study selection using the Preferred Reporting Items for Systematic Reviews and Meta-Analyses (PRISMA) guideline [2]. The inclusion criteria of this study are patients with a history of kidney transplantation and confirmed with COVID-19. The literature selected was written in English in all sources. We excluded all studies that performed transplantation during the recent pandemic time. The type of literature, including epidemiological studies, article reviews, systematic reviews, meta-analysis studies, and case reports, were excluded.

**Fig. 1:**
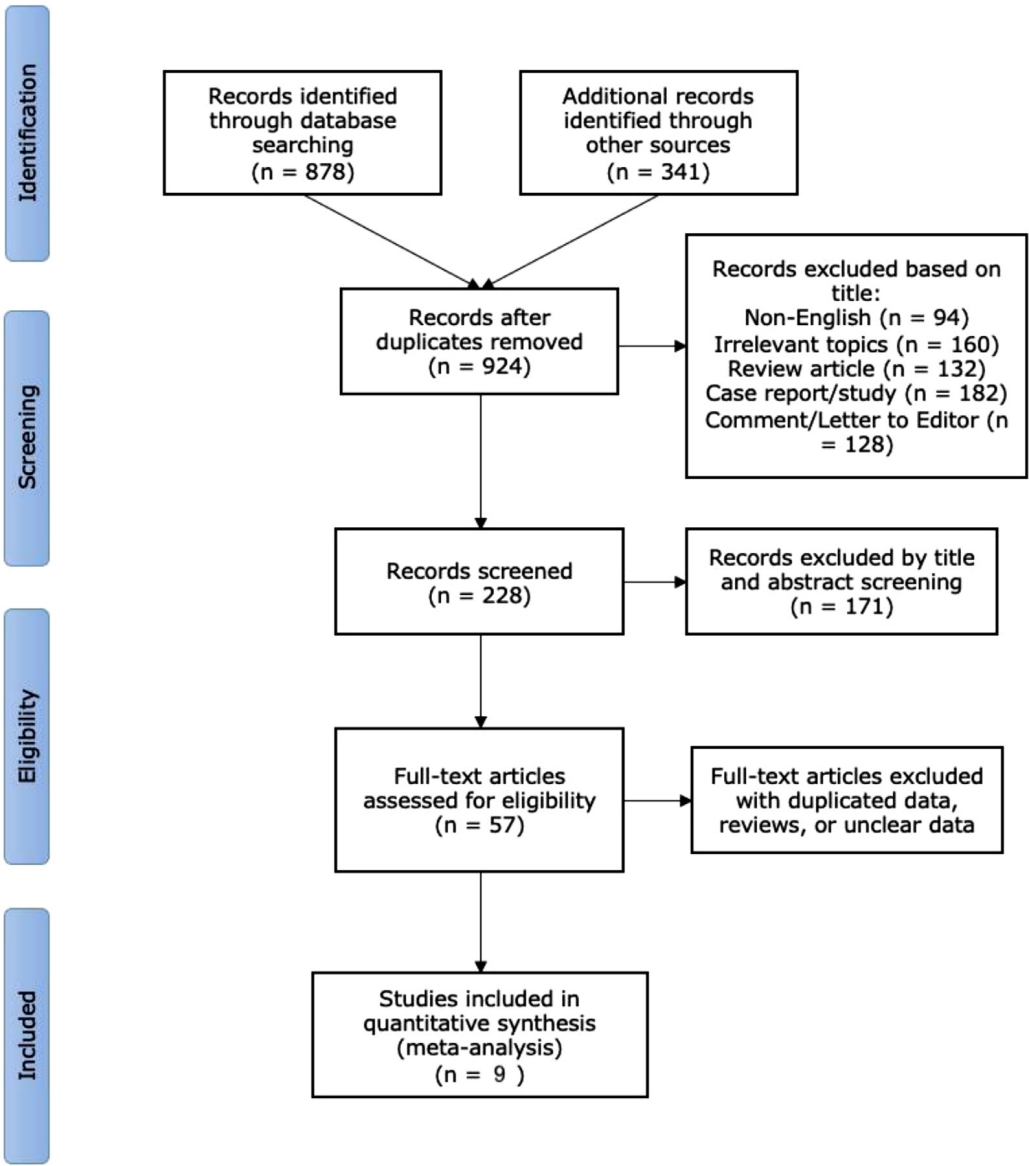
Flowchart of literature search and selection.

The authors performed literature searching using the MeSH terms “kidney transplantation”, “COVID-19″, “SARS-CoV-2″, and “Risk Factor” through electronic databases (Pubmed, Medline, Science Direct, Cochrane Databases, EMBASE, Scopus, EBSCO), published until July 2021. The literature search also used keywords similar to MeSH terms, such as “renal transplant”, “coronavirus infection”, and others. A total of 1219 articles were identified in the database searching for screening. The 10 studies were identified as eligible studies, as shown in Fig. 1. However, we only analyzed nine studies because of duplicated data in different studies with the same main author [5,6].

Finally, the authors performed meta-analyses of 9 published studies [5,[7], [8], [9], [10], [11], [12], [13], [14]]. The information data includes the first author, publication year, study location, study design, total patients, patient characteristics, kidney transplant profile, comorbidities, and Newcastle – Ottawa Scale (NOS).

### Literature analysis

The worse outcomes were defined as in-hospital patients who suffer severe COVID infection, which may lead to death. From that, we analyzed the factors which possibly contributed to the outcomes of the patients and defined them as the predictor factor without any concern for vaccination status and antibody markers.

The meta-analysis was done by Review Manager 5.4 software. All the results were delivered using a forest plot. The variables were compared through mean difference (MD) or odd ratio (OR) with a 95% confidence interval (CI). The outcomes were presented in two groups, worse or good outcomes. The heterogeneity of the studies was measured using I2 with a value of more than 50% is seen as considerable heterogeneity. The significance of the analysis is measured by a p-value less than 0.05 is considered significant. The high heterogeneity analyses among the included studies, patient characteristics, sample size, and laboratory follow-up highlight the cautiousness of applying these results to specific populations.

**Fig. 2:**
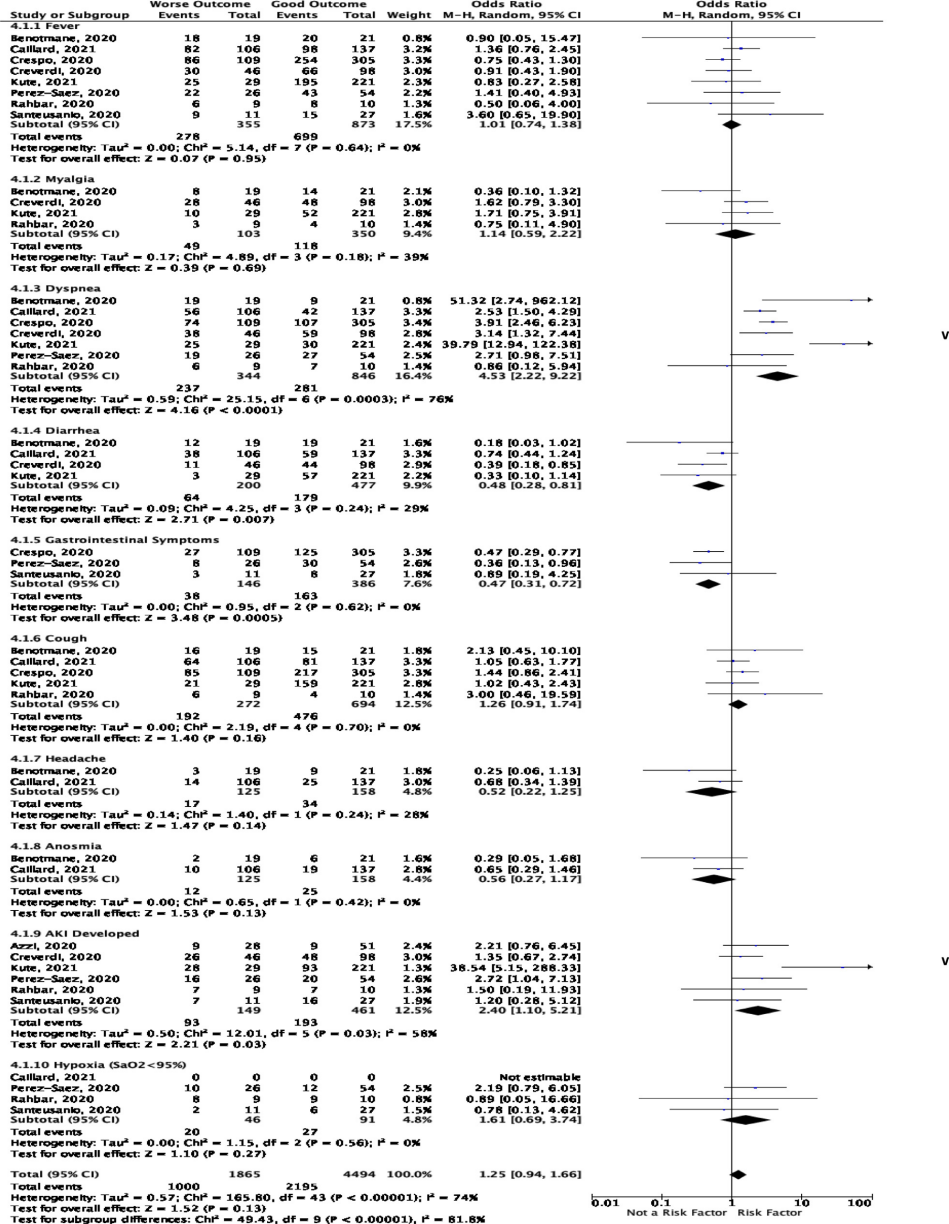
Patients who suffered dyspnea or developed acute kidney injury significantly had a worse outcome. In contrast, diarrhea or other gastrointestinal symptoms were significant inversely.

The meta-analysis was conducted in several categories, such as patient characteristics, kidney profile, patient's comorbid, prior medication, symptoms, and laboratory markers. Each of them has more than one predictor value. After the first forest plot result had been presented, the sample in Fig. 2. In the analysis of the symptoms shown, when the patients were contaminated by coronavirus disease 2019 (COVID-19), they experienced nine symptoms and progression to acute kidney injury (AKI). The analysis showed significant results in symptoms of dyspnea, diarrhea, gastrointestinal symptoms except for diarrhea, and the progression of symptoms to AKI.

The forest plots in symptoms analysis had unusually significant results, both as a risk factor and not a risk factor. There were two symptoms as risk factors, dyspnea and the development of acute kidney injury. Inversely, diarrhea and other gastrointestinal symptoms demonstrated a significant result as not a risk factor, which can be a result of multifactorial and uncontrolled confounding factors. The chance of reducing the value of worsening symptoms was also not performed. The other risk factors, such as fever, myalgia, cough, headache, anosmia, and hypoxia, showed insignificant analysis (*p* > 0.05). Thus, dyspnea and the development of AKI were the only risk factors that were analyzed.

**Fig. 3:**
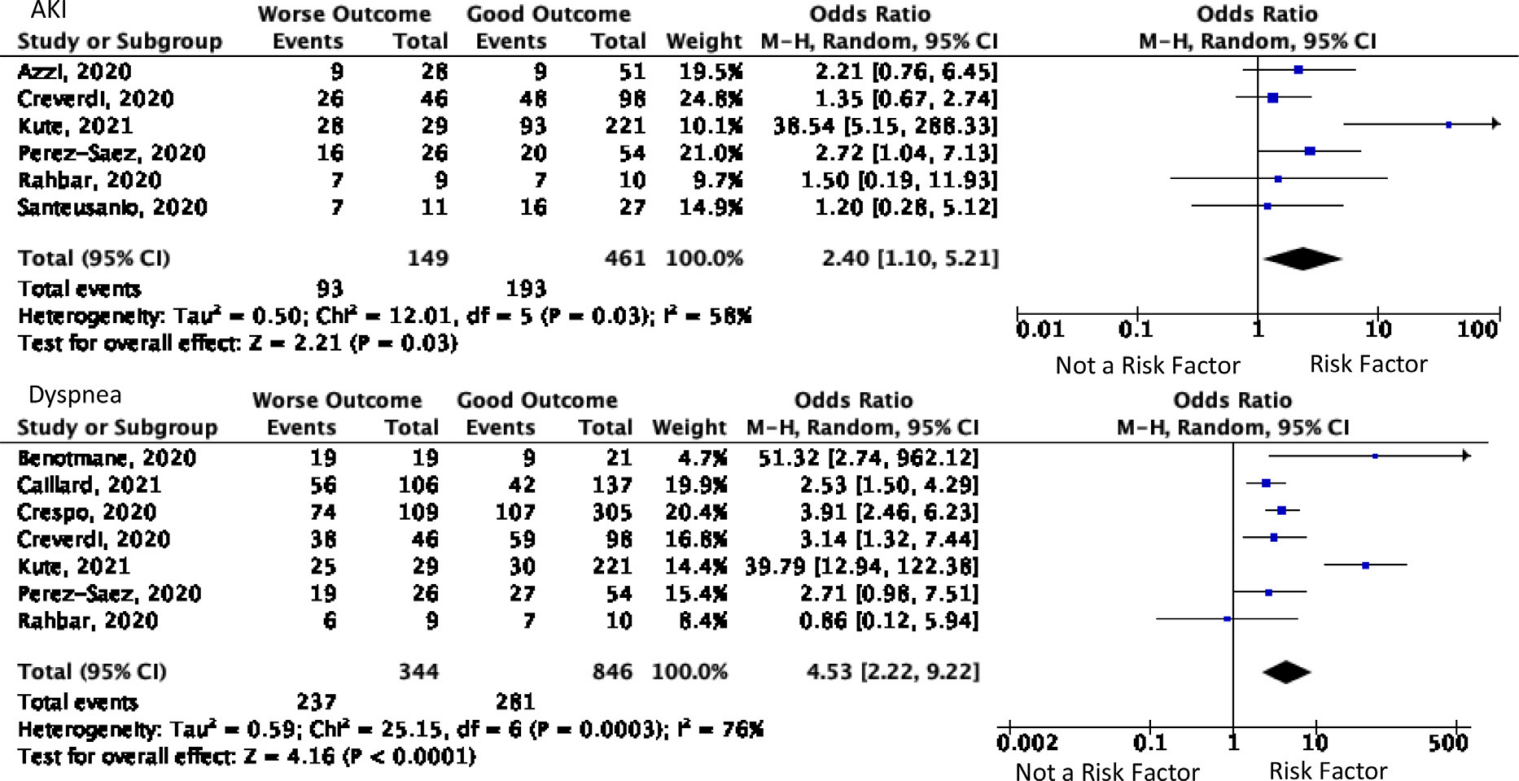
AKI and dyspnea were the significant symptoms with high heterogeneity, I^2^ > 50%.

The isolated analysis of dyspnea and the AKI symptoms could determine the isolated heterogeneity and significance. As shown in Fig. 3, AKI symptoms developed 2.4 times [(95% CI, 1.10–5.21), *p* = 0.03, I2 = 58%] higher in patients with worse outcomes, compared to others on 610 patients in six studies with random analysis due to high of heterogeneity. Moreover, dyspnea has been 4.53 times [(95% CI, 2.22–9.22), *p* < 0.0001, I2 = 76%] more frequently complained of by patients with poor outcomes from seven studies in 1190 patients.

## Conclusion

Our studies demonstrated the fundamental steps to conducting systematic review and meta-analysis studies including the PICOT framework, PRISMA strategy, and comparing the variable in forest plots are mandatory to reduce the bias in the study. The method can guide to generate that many patients had two or multiple risk factors in combination and help to synthesize the data that focus on multi-risk factors. The most significant risk factors for the worse COVID-19 outcomes for kidney transplant patients are dyspnea (*p* < 0.0001), acute kidney injury (*p* = 0.03), and several comorbid and laboratory markers. The limitation of our study was that did not analyze the protective factor, current treatment options, and the significance of the specific drug. Therefore, a further systematic review and meta-analysis of the management pattern relating to COVID-19 in kidney transplants would be necessary.

## Ethics statements

None.

## CRediT authorship contribution statement

**Gede Wirya Kusuma Duarsa:** Conceptualization, Methodology, Writing – original draft, Project administration, Investigation. **Ronald Sugianto:** Methodology, Writing – review & editing, Data curation, Resources, Formal analysis. **I Gusti Agung Ayu Andra Yusari:** Writing – review & editing, Data curation, Resources, Formal analysis. **Pande Made Wisnu Tirtayasa:** Conceptualization, Methodology, Investigation, Visualization. **Gerhard Reinaldi Situmorang:** Conceptualization, Visualization, Validation. **Nur Rasyid:** Conceptualization, Visualization, Validation. **Arry Rodjani:** Conceptualization, Visualization, Validation. **Besut Daryanto:** Visualization, Validation. **Kurnia Penta Seputra:** Visualization, Validation. **Paksi Satyagraha:** Visualization, Validation.

## Declaration of Competing Interest

The authors declare that they have no known competing financial interests or personal relationships that could have appeared to influence the work reported in this paper.

## Data Availability

The data that has been used is confidential.
